# Safety climate in English general practices: workload pressures may compromise safety

**DOI:** 10.1111/jep.12437

**Published:** 2015-08-16

**Authors:** Brian G. Bell, David Reeves, Kate Marsden, Anthony Avery

**Affiliations:** ^1^ Division of Primary CareUniversity of NottinghamNottinghamUK; ^2^ Centre for Primary CareUniversity of ManchesterManchesterUK

**Keywords:** clinical safety, errors, health policy, patient safety, primary care, safety climate

## Abstract

**Objectives:**

Although most health care interactions in the developed world occur in general practice, most of the literature on patient safety has focused on secondary care services. To address this issue, we have constructed a patient safety toolkit for English general practices. We report how practice and respondent characteristics affect scores on our safety climate measure, the PC‐Safequest, and address recent concerns with high levels of workload in English general practices.

**Methods:**

We administered the PC‐Safequest, a 30‐item tool that was designed to measure safety climate in primary care practices, to 335 primary care staff members in 31 practices in England. Practice characteristics, such as list size and deprivation in the area the practice served, and respondent characteristics, such as whether the respondent was a manager, were also collected and used in a multilevel analysis to predict PC‐Safequest scores.

**Results:**

Managers gave their practices significantly higher safety climate scores than did non‐managers. Respondents with more years of experience had a more negative perception of the level of workload in their practice. Practices with more registered patients and in areas of higher deprivation provided lower safety climate scores.

**Conclusions:**

Managers rated their practices more positively on our safety climate measure, so the differences between the perceptions of managers and other staff may need to be reduced in order to build a strong safety culture. Excessive workload for more experienced staff and lower safety climate scores for larger practices may reflect ‘burnout’. Concerns that pressures in primary care could affect patient safety are discussed.

## Introduction

Patient safety has been defined as the ‘avoidance, prevention, and amelioration of adverse outcomes or injuries stemming from the processes of health care’. Although most health care interactions in the developed world occur in general practice with 555 million visits to general practitioners being made annually in the USA [1] and 340 million visits being made annually in the UK [2], most of the literature on patient safety has focused on secondary care services [3]. However, one review of the frequency of error in general practice suggested that between 5 and 80 safety incidents occur per 100 000 consultations [4], in the UK, which would amount to 37–600 incidents everyday, although the wide range on this estimate is an indication of how little is known about the actual level of risk. The potential for errors in general practice is large, but the knowledge base is limited.

One reason may be that general practice is thought of as inherently low risk, so safety is not considered a critical problem. However, serious errors leading to morbidity and mortality do occur in general practice, as shown by previous studies [5, 6]. Understanding the epidemiology of hospital errors was crucial for improving safety in hospitals and gaining public support for efforts to improve safety. There needs to be a similar focus on general practice. In 2011, the American Medical Association's 10‐year report [3] concluded that major gaps remain in our understanding of primary care patient safety with virtually no credible studies on how to improve safety. To address this issue, the National Institute for Health Research School for Primary Care Research (NIHR‐SPCR) in the UK funded a project to construct a patient safety toolkit for English general practices. This toolkit measured several dimensions of patient safety, but our paper will focus on one of these dimensions, namely, safety climate.

Safety climate refers to the components of safety culture [7] that can be measured. Safety culture, in turn, determines how safety is managed by a team or organization. The attitudes, values, perceptions and behaviours, which help to shape the team or organization's commitment to safety, collectively form the team's safety culture [8]. The tool used to measure safety climate in our study was the PC‐Safequest. Practice staff ratings on the tool were summarized and fed back to practices as a means of helping them improve their approach to safety management. Although earlier papers [7, 9] looked at how respondent characteristics were related to PC‐Safequest scores, the authors did not investigate several practice characteristics that might affect responses. Therefore, we have extended this work by considering a wider range of practice characteristics that may affect safety climate scores.

## Methods

### Measures

PC‐Safequest is a 30‐item questionnaire that is designed to measure staff perceptions of safety in primary care practices. The questionnaire is completed by both clinical and non‐clinical staff. Each of the 30 items is measured on a 7‐point scale that ranges from 1 (not at all) to 7 (to a very great extent) and indicates the degree to which each item applies to or characterizes the practice. Higher scores indicate higher perceived practice safety among staff members with the 30 items falling into five dimensions, each measuring a different aspect of safety climate: (i) workload; (ii) communication; (iii) leadership; (iv) teamwork; and (v) safety systems.

We also collected respondent and practice characteristics. Respondent characteristics included gender, whether the respondent worked full‐time or part‐time, number of years of experience in the practice, number of total years of experience in general practices and role. From the role information, we constructed two additional variables, manager/non‐manager and clinician/non‐clinician. Practice characteristics [10] included Quality and Outcomes Framework (QOF) scores (a measure of level of clinical quality [11]), list size (number of patients registered with the practice), the area Index of Multiple Deprivation (IMD) score (which measures social and economic disadvantage [12]) and the percentage of patients over 65 years of age. These practice characteristics were chosen because each one represents stresses that could affect safety climate in a busy practice. All of the practice characteristics were based on figures for 2013 with the exception of deprivation which was calculated in 2010.

### Procedures

We recruited a total of 37 general practices: 9 in Birmingham, 8 in Manchester, 10 in the East Midlands, which included Nottingham and 10 in Southampton. General practices were recruited through the NIHR Primary Care Research Network (PCRN). We tried to ensure that the practice sample was generally representative of English general practices in terms of practice size, demographic characteristics of the practice population and QOF scores. We first recruited sites in Keele, Manchester and the East Midlands, and examined the characteristics of the practices. Then, to obtain a sample that was more representative of English general practices, we asked Birmingham and Southampton to select smaller and more ethnically diverse practices.

After obtaining ethical approval, a recruitment pack was sent to general practitioner (GP) practices via their local PCRN to find out if the practice wanted to be involved in the study. Researchers then met with the practices to discuss the project and answer any questions. Potential participants were informed that their participation was voluntary and that their questionnaire responses would be kept confidential, such that any information fed back to the practice would not allow individual staff members to be identified. Participants were also told that they could withdraw at any time from the study, but none did.

Practice managers registered their practices on a website specifically set up for the project and entered the names of staff who had email accounts. The website then emailed respondents with a link to the questionnaire. Practice managers also printed a letter that invited staff without email accounts to log on to the website to complete the questionnaire, and 1 month later were encouraged to send out automated reminders to staff who had not yet completed the questionnaire. Data were collected through the website, with practice managers receiving reports for their practice, which were shared with staff. These reports compared the practice's results with those of other practices in the study and also broke down the results by role (manager/non‐manager and clinical/non‐clinical).

### Analysis

We applied multilevel modelling to examine whether respondent and practice characteristics were significant predictors of scores on the Safequest questionnaire, so as to account for the nesting of responses within practices. A separate analysis was conducted for scores on each of the five scales (workload, communication, leadership, teamwork and safety systems). A total score, which represented the average value across the five subscales, was also computed for each staff member who completed the questionnaire.

As a first step, each respondent characteristic was tested as a sole predictor in the model. As a second step, those respondent characteristics related to the outcome individually at *P* < 0.01 (to avoid premature exclusion) were used together in a final multilevel model along with the set of practice‐level characteristics. The size of the practice sample meant that power to detect significant relationships with practice‐level characteristics (list size, deprivation score, QOF score and percentage of patients over 65 years of age) was relatively low. Therefore, these predictors were not subjected to individual testing, but were included as a group in the final multilevel model. The Safequest scores and each of the practice‐level predictors were standardized in order to facilitate interpretation of the regression coefficients as standardized beta coefficients. None of the respondent‐level variables were standardized as they were all dichotomous, these coefficients can be interpreted as standardized mean differences between the two levels of the predictor.

Multilevel analysis was undertaken in STATA version 13 using the Xtmixed command with maximum likelihood estimation. Practice mean scores were treated as a random effect and robust (Huber–White) estimates of standard error were used to account for correlated scores between staff within practices. An alpha level of significance of 5% was used except in the first step noted above.

## Results

A total of 335 respondents out of 1150 contacted (29% response rate) completed the questionnaire, which represented 31 out of the 37 (84%) general practices in our sample. With the exception of one variable (full‐time or part‐time employment), which two respondents did not answer, there were no missing data for either the predictors or the dependent variables. Descriptive statistics for each of the predictors are provided in Table 1. The set of practices were close to the English average on all of the practice‐level variables except for list size for which sample practices tended to be larger. Most (83%) of the respondents were female with almost 60% working part‐time and 30% having a managerial position (GPs and practice managers). Clinicians and non‐clinicians were equally represented as were respondents with 10 years or less of experience compared with those with more than 10 years of experience overall. However, about two‐thirds of respondents had 10 or less years of experience in their current practice.

**Table 1:** Descriptive statistics for predictors

Practice‐level predictors*	Mean (standard deviation)
Deprivation score	22.16 (11.43) English average 21.5**
List size	9452 (4618) English average 7041
Qualities and outcomes framework score	975.48 (25.54) English average 961
65 years of age or greater	16.26% (7.00%) English average 16.7%
Respondent‐level predictors	Frequencies (percentages)
Gender	Male 56 (16.7%) Female 279 (83.3%)
FT/PT***	FT 195 (41.4%) PT 138 (58.6%)
Manager?	Manager 102 (30.4%) Non‐manager 233 (69.6%)
Clinician?	Clinician 155 (46.3%) Non‐clinician 180 (53.7%)
Practice service (length of time working in the current position within the current practice)	10 years or less 213 (63.6%) greater than 10 years 122 (36.4%)
Total service (length of time working in primary care in any capacity)	10 years or less 171 (51%) greater than 10 years 164 (49%)

*The means and standard deviations were weighted by the list size of the practice.

**No standard deviation was provided.

***Missing responses for two participants.

Table 2 shows the means and standard deviations for each of the Safequest scales. Mean scores ranged from 4.2 to 5.5 indicating that staff members generally thought that their practices achieved safety goals to a ‘moderate’ (score of 4), considerable (score of 5) or ‘great’ (score of 6) extent.

**Table 2:** Descriptive statistics for Safequest scales

Scale	Mean (standard deviation)
Workload	4.2 (1.2)
Communication	4.7 (1.4)
Leadership	5.5 (1.3)
Teamwork	5.3 (1.2)
Safety systems	5.5 (1.2)
Total	5.1 (1.0)

**Figure 1:**
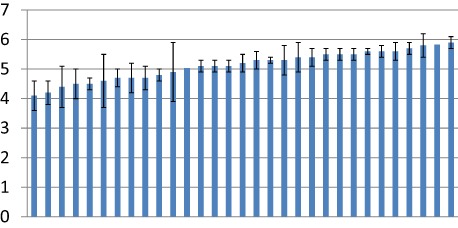
Mean total scores for 31 practices.

The variability between practices is shown in Fig. 1 for the total PC‐Safequest score. Although these graphs suggest there was quite a bit of variability between practices, the intra‐class correlation coefficients in Table 3 reveal little clustering within practices for the Communication and Safety Systems scales, which are poorer at discriminating between practices than the other scales. The practice mean reliability coefficients were all less than 0.7, which implies that for the average practice in our study, none of the Safequest scale scores met the accepted standard for reliability.

**Table 3:** Intra‐class correlation coefficients and reliability coefficients

Scale	ICC (95% CI)	Practice mean reliability coefficient*
Workload	0.12 (0.02 to 0.22)	0.59
Communication	0.04 (0.00 to 0.11)	0.31
Leadership	0.13 (0.03 to 0.24)	0.62
Teamwork	0.14 (0.03 to 0.25)	0.63
Safety systems	0.05 (0.00 to 0.12)	0.36
Total	0.10 (0.00 to 0.19)	0.53

*Reliability coefficients were based on approximately 11 staff in each practice.

The results from the multi‐level analysis are shown in Table 4. Whether the respondent was a manager or not was a significant predictor for all of the scales except workload. Managers (GPs and practice managers) gave their practices higher scores on the safety climate scales than did non‐managers with Table 5 showing the difference in mean scores between managers and non‐managers. For the workload scale, the total time spent working in general practices was a significant predictor, a longer time in service was associated with more negative perceptions of workload.

**Table 4:** Statistically significant predictors of Safequest scores

Safequest scale	Predictor	Regression coefficient^a^ (95% CI)	Z
Workload	Total time in service Practice list size	−0.21 (−0.37 to −0.04) −0.18 (−0.31 to −0.05)	−2.46* −2.68**
Communication	Manager role Percentage over 65 Practice list size Deprivation score	0.84 (0.58 to 1.10) −0.13 (−0.25 to −0.02) −0.25 (−0.34 to −0.16) −0.21 (−0.31 to −0.11)	6.29** −2.25* −5.41** −3.90**
Leadership	Manager role Practice list size Deprivation score	0.46 (0.21 to 0.72) −0.22 (−0.34 to −0.09) −0.28 (−0.46 to −0.10)	3.62** −3.40** −3.04**
Teamwork	Manager role Practice list size Deprivation score	0.52 (0.28 to 0.76) −0.35 (−0.50 to −0.20) −0.27 (−0.45 to −0.09)	4.26** −4.46** −2.98**
Safety systems	Manager role Practice list size	0.57 (0.31 to 0.83) −0.20 (−0.32 to −0.07)	4.34** −3.07**
Total Score	Manager role Practice list size Deprivation score	0.65 (0.40 to 0.91) −0.29 (−0.40 to −0.18) −0.26 (−0.39 to −0.14)	5.00** −5.02** −4.10**

*P < .05.

**P < .01.

a Regression coefficients for dichotomous predictors are expressed as standardized mean differences; for continuous predictors as standardized beta coefficients.

**Table 5:** Mean differences between managers and non‐managers on Safequest scales

Scale	Difference between mean scores of managers and non‐managers (95% CI in parentheses)
Workload	0.2 (−0.13–0.44)
Communication	1.1 (0.90–1.46)
Leadership	0.6 (0.30–0.82)
Teamwork	0.6 (0.33–0.84)
Safety systems	0.7 (0.48–0.94)
Total	0.7 (0.44–0.87)

Regarding the practice‐level predictors, list size was significantly related to scores on all of the scales. Practices with more registered patients had lower safety climate scores. The deprivation score was also a significant predictor for all of the scales except for the workload and safety systems measures: practices in areas with higher levels of deprivation had lower safety climate scores. Although not shown in the table, level of deprivation was also a marginally significant predictor of both workload and safety systems scores (0.058 for workload and 0.059 for safety systems). The percentage of registered patients over 65 was significantly related to scores on the communication scale, with practices that had a larger percentage of older patients being rated lower on this scale. Beta coefficients for all of the practice‐level predictors were mostly between 0.2 and 0.3, suggesting low to moderate relationships. The only practice‐level predictor that showed no significant relationships with any Safequest scale was the QOF score, which may have been due to the lack of variability between practices. The mean in Table 1 shows that a large number of practices obtained a value close to the maximum (1000), with the lowest score being 883.

## Discussion

Managers rated their practices significantly higher on our safety climate measure, PC‐Safequest, than did other employees. We also found that the number of years spent working in general practices was a significant predictor of workload scores. This relationship, in which more experience is associated with more negative perceptions of workload, may reflect ‘burnout’ among more experienced staff.

Several practice‐level predictors were significantly, although modestly, associated with PC‐Safequest scores. Practices in more deprived areas and those with a larger number of registered patients fared more poorly on most of the scales. We also found that the percentage of older patients, those over 65 years of age, was negatively related to communication scores. Documenting the relationship between external pressures and safety climate, which we have done in this paper, may raise awareness of this problem and improve patient safety in general practice.

Notwithstanding these relationships, we found that staff agreement on how they rated the Safequest scales was not much higher within practices than it was across practices, such that the instrument was poor at discriminating between practices in terms of reported safety climate. Indeed, for a typical practice in our study, with 11 responding staff, practice mean scores on all of the Safequest scales would fail to meet accepted levels of reliability. Thus, in this study, Safequest did not perform well as an instrument for producing a ‘snapshot’ of the safety climate for a particular practice at a particular time, though we should not conclude from this that it has no value as a means for stimulating practices to reflect on and improve their safety management.

In general, our results support what other researchers [7] found for a sample of Scottish general practices. These authors reported that managers rated their practices more highly on all of the Safequest scales, except for the workload scale, and suggested that differences between the perceptions of managers and other staff may need to be reduced in order to build a strong safety culture. In the Scottish study [7], the number of years of experience in the current practice, rather than in all practices, was a significant predictor of lower workload scores, but this still supports our contention that more experienced staff may be experiencing ‘burnout’.

We should again note that most of the relationships were modest in size, which reflects other work that has been done in this area [9]. Practice‐level predictors produced smaller beta coefficients than the manager variable, which was measured at the respondent level. A recent study of US federal agencies found that organizational‐level variables were less important than variables measured at the individual level in predicting perceived organizational performance [13].

One of the strengths of our study is the large number of respondents who participated and the low rate of missing data among those who completed the survey. However, although 84% of the practices provided data, only 29% of potential respondents participated in the study. Self‐report measures depend on the respondent being willing to share their opinions. Anonymity was preserved by only providing practice‐level feedback and having individual respondents use a website to respond, but the low response rate may have been due to concerns with confidentiality. In the earlier Safequest study with a Scottish sample [7], only 25% of contacted practices participated, although 84% of the potential respondents completed the questionnaire. These response rates are effectively reversed compared with ours, yet the main findings around predictors of Safequest scores were essentially the same, suggesting that our findings are robust against non‐response bias.

We were also able to sample four different parts of the country: Birmingham, Manchester, the East Midlands and Southampton, with our sample of practices matching the English average on several demographic variables. Unfortunately, our sample contained very few men (17%), although a similar gender imbalance was found in another study with the PC‐Safequest [7]. A final strength was the use of a well‐validated safety climate measure that was specifically designed for use in primary care.

Future research is needed to link safety climate with patient outcomes in primary care, especially ‘hard’ outcomes such as hospitalizations, emergency department visits and mortality. Research is also needed on how a safety culture can be built and maintained in primary care, and the obstacles that may prevent this from happening. The pressures on general practices may create safety problems that need to be addressed in future studies, which bring us to one aspect of the study that should be emphasized, namely workload.

Respondents in our study provided lower scores on the workload scale than on any other scale. We also found that both list size and the percentage of years working in general practices were negatively associated with these scores. As the British Medical Association pointed out recently [14], GP practices have been under enormous pressure with the number of consultations in England increasing from 300 million to 340 million in the last 5 years. At the same time, resources for primary care have fallen with 74% of GPs believing that their workload was not manageable [14].

This perception among GPs coincides with our suggestion that ‘burnout’ may be responsible for more experienced staff providing more negative perceptions of workload, which could compromise patient safety. Unfortunately, task demands were a contributing factor in almost half of the adverse events and near misses that were found in one recent study that was conducted in primary care [15]. The items on the workload scale specifically link compromises to patient safety with high workload. One item asks staff whether ‘Team members always have enough time to complete tasks safely’ and another asks whether ‘The level of staffing in the practice is sufficient to manage the workload safely’. Therefore, our findings may reflect the negative, cumulative effects of high workload on patient safety in primary care and provides a timely reminder that excessive workload remains one of the major challenges facing primary care practices in England.

## Conflict of interest

The authors declare no conflict of interest.

## Author contributions

BB wrote the paper and analysed the data, DR supervised the analysis and provided statistical advice, KM helped to run the study and made valuable suggestions on the manuscript, and AA supervised the study and provided comments on preliminary drafts. All authors read and approved the final manuscript.
